# Expression of toll-like receptors in human limbal and conjunctival epithelial cells

**Published:** 2007-06-08

**Authors:** Jing Li, JiangBo Shen, Roger W. Beuerman

**Affiliations:** 1 Singapore Eye Research Institute, National University of Singapore, Singapore; 2 Department of Ophthalmology, Yong Loo Lin School of Medicine, National University of Singapore, Singapore; 3 Division of Chemical & Biomedical Engineering, Nanyang Technological University, Singapore

## Abstract

**Purpose:**

To determine the expression and function of toll-like receptors (TLRs) in human conjunctival, limbal and corneal epithelial cells.

**Methods:**

Expression of TLRs was examined by real-time polymerase chain reaction, immunohistochemistry, and western blot analysis in human conjunctival, corneal and limbal epithelial cells and tissues. Ligand-stimulated nuclear factor κB activation; interleukin 6 and interleukin 8 protein secretion was measured in the cultured conjunctival and limbal epithelial cells by ELISA analysis.

**Results:**

Expression of TLR1, 2, 3, 5, and 6 was found in all conjunctival and limbal epithelial cell samples analyzed by real time PCR and western blot. TLR4 and TLR9 transcripts were undetectable in some samples by real-time PCR. TLR7, 8 and 10 transcripts were not detected by real time PCR in any of the samples tested. TLR1, 2, 3, 4, and 5 proteins were found in conjunctival, limbal and corneal epithelium by immunohistochemistry. Cultured conjunctival epithelial cells expressed significantly lower levels of TLRs than uncultured conjunctival cells obtained by applying nitrocellulose paper to the bulbar conjunctival surface. Cultured limbal and conjunctival cells responded to stimulation by polyriboinosinic polyribocytidylic acid (poly[I:C]), palmitoyl-3-cysteine-serine-lysine-4 (Pam3CSK) and flagellin with increased secretion of IL-6 and IL-8 and the activation of NFκB. Peptidoglycans (PGN) and CpG DNA caused increased NFκB activity; however, only conjunctival epithelial cells showed increased cytokine secretion. Lipoteichoic acid (LTA) or lipopolysacchride (LPS) did not change cytokine secretion or NFκB levels in either cell type.

**Conclusions:**

The TLRs found in human conjunctival and limbal epithelial cells provide a basis for responses to many common ocular pathogens. Although the mRNA and protein for TLR4 and TLR2 was found, neither conjunctival or limbal cells in culture responded to LPS or LTA stimulation.

## Introduction

The rapidly deployable innate immune system of the ocular surface provides an early response against microbial invasion. Important components of the ocular surface's innate immune system include the physical barrier to pathogen entry, the presence of antimicrobial molecules in the tear film, and pattern recognition receptors such as the cellular toll-like receptors (TLR). Activation of these receptors promotes the release of cytokines, chemokines, and other molecules, which participate in inflammatory responses and the activation of the adaptive immune system.

Toll-like receptors are a family of evolutionarily conserved membrane receptors first identified in *Drosophila* [1]. In humans, there are 11 members of the TLR family (TLR1-TLR11) which are found in a wide variety of cells including epithelial cells and those from the immune system such as neutrophils, macrophages and dendritic cells [2]. Generally, a TLR binds to a specific molecular pattern presented from a pathogen such as bacteria, viruses, fungi, or parasites [2]. However, since each pathogen produces more than one kind of pattern molecule, there is considerable redundancy in TLR-mediated pathogen recognition responses. For example, viral RNA is the ligand for TLR3, 7 and 8. Bacteria genomic DNA is recognized by TLR9; bacteria flagella protein, flagellin, is recognized by TLR5 [3]. A cell wall component, peptidoglycan (PGN) of gram-positive bacteria is the preferred ligand of the TLR2 homodimer, and lipopolysaccharides (LPS) of gram-negative bacteria is recognized by TLR4 [4]. The binding and activation of LPS to TLR4 needs three additional components: MD2, a membrane protein whose association with TLR4 is required for the binding of LPS [5]; LPS-binding protein (LBP), which extracts LPS monomers from the aggregated form [6,7]; and CD14, which transfers LPS to the TLR4/MD2 transmembrane co-receptor, which then triggers the downstream molecular events [2]. The TLR1/TLR2 heterodimer recognizes tri-acyl lipopeptides while TLR2/TLR6 recognizes lipotechoic acid (LTA) and di-acyl lipopeptides produced by mycoplasma [8-10]. Binding of ligands to TLRs leads to the activation of a complex signaling cascade of events including the activation of the transcription factor NFκB and an increased expression of inflammatory cytokines [11,12].

When a pathogen contacts the ocular surface, the tears provide a medium to disseminate the ensuing signaling molecules over the corneal, conjunctival and limbal epithelial cells constituting the ocular surface. As TLRs are a critical component of the innate immune system, the distribution of their response capabilities across the ocular surface may be expected to cover the various pathogen signals. However, the expression of TLRs in limbal and conjunctival epithelial cells is largely unknown except for a recent study reporting the expression of TLR2, 4 and 9 in healthy and allergic human conjunctiva by RT-PCR and immunohistochemistry [13]. In human cornea, multiple groups have reported the expression of TLR3 [14,15], TLR4 [16,17], and TLR5 [18] mRNA and proteins. A recent study showed that herpes simplex virus, HSVI, induced the expression of TLR7 in corneal epithelium [19]. Similarly, TLR2 [20], 4 [20], and 9 [20,21] were identified in the mouse cornea. However, there is a controversy as to whether TLR2 and TLR4 found in human cornea epithelial cells respond to LPS stimulation [14,16,17]. One of the studies showed that the presence of LPS stimulated the secretion of IL-6 and IL-8 in a human corneal epithelial cell line, which could be further inhibited by adding antibody against CD14, clearly suggested a functional TLR4 in these cells [16]. However, another group showed that LPS had no effect on TLR4 surface distribution or IL-6 and IL-8 secretion in either primary cultured or immortalized human corneal epithelial cells [14,17]. A more recent report showed that the addition of tear LBP and CD14 were required for LPS-stimulated IL-6 and IL-8 secretion in corneal epithelial cells [22].

In this study, we examined the expression of TLRs1-10 in human conjunctival, limbal and corneal epithelial cells by real time PCR, immunohistochemistry, and western blot analysis. TLR function was examined by determining the activation of NFκB and the secretion of IL-6 and IL-8 in primary cultured human limbal and conjunctival epithelial cells in the presence of ligands specific to each TLR.

## Methods

### Reagents

LPS, isolated from *Pseudomonas aeruginosa* and *E. coli*, and LTA, isolated from *Staphylococcus aureus*, were purchased from Sigma (Sigma Aldrich, Singapore) and were used as ligands for TLR4 and TLR2/TLR6 at concentrations from 10 ng/ml to 10 μg/ml [23], respectively. Flagellin protein (greater than or equal to 98% pure by SDS-PAGE), isolated from *Salmonella typhimurium*, was purchased from Alexis (San Diego, CA) and was used to stimulate TLR5 at the concentration of 2 μg/ml [3,18]. Peptidoglycan (PGN), isolated from *Bacillus subtilis*, was purchased from Fluka (Sigma Aldrich) and was used as the ligand for TLR2 homodimer at the concentration of 10 μg/ml [23]. Polyriboinosinic polyribocytidylic acid (poly[I:C]) was purchased from Amersham (Amersham Biosciences, Piscataway, NJ) and was used as the ligand of TLR3 at the concentration of 25 μg/ml [14,15,24]. Synthetic triacylated lipoprotein analog palmitoyl-3-cysteine-serine-lysine-4 (Pam3CSK), was purchased from InvivoGen (San Diego, CA) and was used as the ligand for TLR1/2 heterodimer at the concentration of 250 ng/ml [25]. Sequences and backbone of CpG DNA phosphorothioate-1668 (CpG DNA; TCC ATG ACG TTC CTG ATG CT) and phosphodiester-1668 (CpG control; TTC ATG ACG TTC CTG ATG CT) was synthesized by Research Biolabs (Singapore) and used as the ligand and control for TLR9 at 1 μM [20,26]. The sequences marked in red denote the typical CpG motif. Carrier-free recombinant human CD14 and LBP protein was purchased from R&D (Research & Diagnostic Systems, Minneapolis, MN).

### Use of human tissues and cells

Human cadaver conjunctival tissues were obtained from the Singapore Eye Bank and used for the isolation and culture of conjunctival epithelial cells within 16 h of death. All donors were males aged from 51-68 years of age with an average of 58 years. Corneoscleral rims, remaining from corneal transplantations at the Singapore National Eye Center, were used for the isolation and culture of limbal epithelial cells within two to seven days after death. During this time, the tissues were kept in chondroitin sulfate/dextran corneal storage media (Optisol^TM^-GS obtained from Bausch & Lomb, St. Louis, MO) at 4 °C. The average age of the corneoscleral rim donors was 72 years (range 61-86 years). Cadaver cornea tissues used for laser micro-dissection of corneal epithelial cells were obtained from Sri Lanka International Eye Bank through the Singapore Eye Bank and were used within 24 h of death. The average age of the cornea tissue donors was 63 years (range 51-72 years). Sterile nitrocellulose paper was used to collect conjunctival epithelial cells from six healthy volunteers (four males and two females, age ranges from 28-40 years with an average of 35 years) with no ocular surface abnormalities. After one drop of 0.5% amethocaine hydrochloride for topical anesthesia, a 2 mm by 3 mm sterile nitrocellulose paper (Millipore, Billerica, MA) was applied to the bulbar conjunctival surface with a blunt, smooth-tipped forcep [27]. Area with visible blood vessels was avoided. The paper was carefully removed two to three s later and was immersed in 1 ml of Trizol reagent for immediate RNA extraction. All protocols adhered to the tenets of the Declaration of Helsinki and were reviewed and approved by the Ethics Committee (IRB) of Singapore Eye Research Institute and a signed consent was obtained from each informed participant.

### Laser micro-dissection of corneal epithelial cells

Laser microdissection was used to obtain full thickness corneal epithelium. Briefly, human cadaver corneal tissue within 24 h postmortem was cut and embedded in OCT (Sakura Finetel, Torrance, CA) upon arrival and kept at -80 °C. Cornea epithelial cells were obtained by laser microdissection using the PALM Combi system (PALM Microlaser Technologies, Germany). Immediately before PALM dissection, the embedded tissue was removed from -80 °C storage, quickly cut at 10 μm on a cryostat, fixed and stained in: 70% ethanol for 30 s, DEPC water for three quick dips, hematoxylin 10 μl/section for 20 s, DEPC water for four quick dips, 95% ethanol for 30 s, and 100% ethanol for 30 s. To minimize RNA degradation during the process, all solutions were made in DEPC-treated water. After dehydration, the slide was mounted on the PALM microscope stage and corneal epithelial cells were collected into tubes containing 20 μl of Trizol.

### Isolation and cultivation of limbal and conjunctival epithelial cells

Limbal epithelial cells were isolated from the corneoscleral rim which remained after the central cornea was removed for corneal transplantation at the Singapore National Eye Centre [28]. Briefly, the tissue was digested by 1.2 IU/ml dispase II at 37 °C for three h after being trimmed and washed in antibiotic solution. Loosened epithelial sheets were removed with a cell scraper and were separated into single cells by trypsin digestion. Cells were plated at 10^4^ cells/cm^2^ in cell culture dishes containing mitomycin C (MMC)-treated 3T3 feeder layer (pretreated with 4 μg/ml MMC for two h at 37 °C and plated at a density of 2.2x10^4^ cells/cm^2^ 16-24 h before using) in supplement hormonal epithelial medium (SHEM) [29]. The medium contain an equal volume of DMEM and Ham's F12 supplemented with 5% FBS, 5 μg/ml insulin, 5 μg/ml transferrin, 5 ng/ml sodium selenite, 2.5 u g/ml human recombinant epidermal growth factor (EGF), 8.4 ng/ml cholera toxin, 0.5% dimethyl sulfoxide (DMSO), 0.5 μg/ml hydrocortisone, 50 μg/ml gentamicin, 1.25 μg/ml amphotericin-B, and 5 mM HEPES. Only P_0_ cells were used in this study.

Conjunctival epithelial cells were isolated from cadaver conjunctival tissues by a similar procedure except that they were re-suspended and grown in serum-free keratinocyte growth medium (KGM) supplemented with bovine pituitary extract (BPE), human recombinant epidermal growth factor (EGF), insulin, hydrocortisone, and gentamicin/amphotericin-B (CC-4131 from Cambrex, Walkersville, MD). P_0_-P_1_ cells were used in this study.

### Analysis of toll-like receptor gene expression

RNA was extracted using Trizol reagent (Invitrogen, Singapore) and was reverse transcribed into cDNAs using RTIII (Invitrogen). Gene expression was determined by Taqman gene expression analysis (Applied Biosystems, Singapore) using 250 ng of cDNA in a reaction of 25 μl. The assay IDs of each TLR gene are: TLR1: Hs00413978_m1; TLR2: Hs00152932_m1; TLR3: Hs00152933_m1; TLR4: Hs00152939_m; TLR5: Hs00152825_m1; TLR6: HS00271977_m1; TLR7: Hs00152971_m1; TLR8: Hs00152972_m1; TLR9: Hs00152973_m1; and TLR10: Hs00374069_g1. β-Actin was used as the internal control. Human spleen cDNA (Ambion, Applied Biosystems, Singapore) was used as a positive control for the detection of TLR7, TLR8 and TLR10 expression. For each pair of primers and samples, triplicate wells were used. Negative controls included H_2_O and a RT control, which consisted of the mixture of the RT reaction without reverse transcriptase. Delta C_t_ (ΔC_t_) was calculated by subtracting the C_t_ of β-actin from the C_t_ of the targeted gene. The uncultured conjunctival epithelial cell sample was chosen as the calibrator to compare the relative abundance of each TLR gene transcript among different samples. The fold change in other samples was determined by the formula 2^(-ΔΔC^_t_^)^, where ΔΔC_t_=ΔC_tsample_-ΔC_tcalibrator_. Data were expressed as the mean±SEM and analyzed by ANOVA. The ΔC_t_ of each gene among different cell types were compared by the Fisher least significant difference (LSD) test. A probability level of p<0.05 was considered as statistically significant.

### Western blot analysis

Cultured P_1_ conjunctival and P_0_ limbal epithelial cells were lysed in radioimmunoprecipitation (RIPA) buffer containing 10 mM Tris pH7.5, 150 mM NaCl, 1% sodium deoxycholate, 1% Triton X-100, 1 mM EDTA, and a protease inhibitor cocktail (Roche Diagnostics Asia Pacific, Singapore). Total lysates (40 μg) were loaded on SDS-PAGE, transferred to nitrocellulose paper (Bio-Rad), and blotted with anti-TLR antibodies. Goat anti-TLR1 antibody was purchased from R&D Systems (Minneapolis, MN) and was used at the concentration of 2 ng/lane. Monoclonal anti-TLR2 and anti-TLR3 antibodies were purchased from Imgenex (San Diego, CA) and used at a dilution of 1:100. Rabbit anti-TLR4, rabbit anti-TLR5, and goat anti-TLR6 antibodies were purchased from Santa Cruz Biotechnology (Santa Cruz, CA) and used at a dilution of 1:200. Monoclonal anti-TLR9 antibody purchased from Abcam (Cambridge, UK) was used at a concentration of 1 μg/ml. All antibodies were incubated with substrate overnight at 4 °C and blotted with specific horseradish peroxidase conjugated secondary antibodies purchased from Santa Cruz Biotechnology (1:2000 for anti-rabbit antibody sc-2030, 1:2000 for anti-mouse antibody sc-2005, and 1:5000 for anti-goat antibody sc-2350). The membrane was developed with SuperSignal chemiluminescent substrates from Pierce Biotechnology (Rockford, IL).

### Immunohistochemistry

OCT embedded human tissue was cut at 5 μm and fixed in cold methanol at -20 °C for 15 min. Monoclonal anti-TLR1 antibody (Imgenex), rabbit anti-TLR4 antibody (Santa Cruz) at a dilution of 1:100, goat anti-TLR2, goat anti-TLR5, goat anti-TLR9 antibodies (Santa Cruz) at 1:50 dilution, goat anti-TLR3, and goat anti-TLR6 at 1:200 dilution were used and incubated overnight at 4 °C with 4% BSA/PBS as the blocking reagent. Alexa Fluor 488 conjugated secondary antibody was used at 1:2000 and incubated at RT for one h for visualization (Molecular Probes, Invitrogen). In control tissues, the primary antibody was replaced by the serum corresponding to the animal species from which the primary antibody was raised. VectaShield mounting medium containing DAPI was used (Vector Lab, Burlingame, CA) to counter-stain nuclei.

### NFκB activity analysis

Eighty percent (80%) confluent P_1_ conjunctival and limbal epithelial cells were stimulated with each ligand for two, five, and eight h in six well plates. After stimulation, the cells were washed with cold PBS and the nuclear fraction was extracted. The activities of p65 and p50 subunits of NFκB were measured by ELISA analysis using 96 well plates precoated with NFκB-binding DNA consensus sequence (Pierce, Rockford, IL). Only the active form of p65 and p50 binds to the immobilized DNA sequence and the bound protein is subsequently detected by specific primary antibody against p65 and p50 followed by HRP conjugated secondary antibody. The chemiluminescence signal was measured by the Tecan GeniosPro microplate reader (Tecan Asia, Singapore). Data were expressed as the mean±SE and analyzed by ANOVA coupled with Fisher LSD test. A probability level of p<0.05 was considered as statistically significant.

### Cytokine expression and secretion analysis

IL-6 and IL-8 gene expressions were analyzed by real time PCR using the Taqman gene expression system as described above. β-Actin was used as the internal control. IL-6 and IL-8 protein in the culture supernatant was quantified by a sandwiched ELISA analysis (BD Pharmingen, San Diego, CA). Briefly, P_1_ cells were incubated with specific ligands in supplement hormonal epithelial medium (SHEM; for limbal epithelial cells) or KGM (for conjunctival epithelial cells) in 24 well tissue culture plates at the density of 8x10^4^ cells/well for 24 h before the supernatant was harvested. Microtiter plates coated with IL-6 or IL-8 antibodies were incubated with standards and samples. Detection was achieved using a biotinylated IL-6 or IL-8 antibody together with an avidin-horseradish peroxidase conjugate. Color was developed by using 3,3',5,5'-tetramethylbenzidine (TMB) and read at 450 nm on a microplate reader (Tecan Asia, Singapore). Data were expressed as the mean±SE and analyzed by ANOVA and the Fisher LSD test. A probability level of p<0.05 was considered statistically significant.

## Results

### Toll-like receptor gene expression in conjunctival, corneal and limbal epithelial cells

*TLR*1-10 gene expression in normal bulbar conjunctival epithelial cells (removed by nitrocellulose paper; n=5), corneal epithelial cells (removed by PALM laser microdissection; n=2; referred to as uncultured cells), and six different primary cultured human conjunctival and limbal epithelial cell samples of each (isolated from different donors) was determined by Taqman real-time PCR analysis. Table 1 shows the average ΔC_t_ of each *TLR* transcript tested in these cell samples. Table 2 shows the fold differences of each *TLR* gene level expressed in different samples in comparison with uncultured conjunctival epithelial cells.

**Table 1:** Toll-like receptor1-10 gene expression by Taqman real time PCR analysis.

**Gene**	**Corneal epithelial cells (n=2)**	**Conjunctival epithelial cells (n=5)**	**Cultured limbal cells (n=6)**	**Cultured conjunctival cells (n=6)**
*TLR1*	7.64±1.00	6.33±0.54#	9.42±0.58*	12.78±0.58
*TLR2*	8.17±1.02	7.86±0.65#	7.06±0.72*	12.26±0.72
*TLR3*	5.55±0.93	4.42±0.59#	11.51±0.66	13.35±0.66
*TLR4*	NA	5.36±0.63#	12.69±1.78	15.56±3.06
			(n=5)	(n=4)
*TLR5*	4.75±0.92	4.91±0.58#	9.12±0.65	10.66±0.53
*TLR6*	11.40±1.27	13.28±0.80#	16.53±0.90	18.20±0.80
*TLR7*	NA	NA	ND	ND
*TLR8*	NA	NA	ND	ND
*TLR9*	ND	12.53±1.28	14.86±0.21	15.42±1.81
		(n=3)		(n=4)
*TLR10*	NA	NA	ND	ND

ΔC_t_ of *TLR1-10* gene transcripts. ΔC_t_ of each transcript was calculated by subtracting the C_t_ of β-actin from the C_t_ of target *TLR*. N refers to the number of different samples analyzed. Triplicated PCR analysis was carried out for each sample. The data presented is the average ΔC_t_±SEM. Corneal epithelial cells were collected by laser microdissection. Conjunctival epithelial cells were collected by nitrocellulose paper. ND: nondetectable. NA: data not available due to limited amount of RNA obtained from the samples. The asterisk denotes a p<0.05 when cultured conjunctival cells compared to cultured limbal epithelial cells. The sharp (hash mark) denotes a p<0.05 when cultured conjunctival epithelial cells compared to non-cultured conjunctival epithelial cells collected by nitrocellulose paper.

**Table 2:** Fold difference of each toll-like receptor gene expression among different samples in comparison with uncultured conjunctival epithelial cells.

**Gene**	**Corneal epithelial cells**	**Conjunctival epithelial cells**	**Cultured limbal cells**	**Cultured conjunctival cells**
*TLR1*	0.403±0.046	1.01±0.12	0.119±0.04	0.011±0.01
*TLR2*	0.810±0.14	1.04±0.13	1.745±0.10	0.048±0.01
*TLR3*	0.461±0.15	1.09±0.15	0.007±0.02	0.002±0.01
*TLR4*	ND	1.01±0.13	0.006±0.00	0.001±0.00
*TLR5*	1.117±0.14	1.06±0.14	0.054±0.01	0.019±0.01
*TLR6*	3.681?0.25	1.01±0.08	0.102±0.00	0.033±0.01
*TLR9*	ND	1.01±0.02	0.200±0.00	0.131±0.03

The calculation of the fold difference was described in Methods. Corneal epithelial cells were collected by laser microdissection. Conjunctival epithelial cells were collected by nitrocellulose paper. ND: nondetectable.

*TLR1, 2, 3, 5*, and *6* gene transcripts were detected in all cell samples tested. Uncultured conjunctival epithelial cells expressed higher levels of each of these *TLR* genes when compared to primary cultured conjunctival epithelial cells. *TLR1* and *TLR2* transcripts were found to be more abundant in cultured limbal epithelial cells than cultured conjunctival epithelial cells (p<0.05). No significant difference in ΔC_t_ was observed between uncultured corneal and conjunctival epithelial cell samples for *TLR 1, 2, 3, 5*, or *6* gene transcripts.

The *TLR9* gene transcript was detected in all cultured limbal cell samples. However, only three out of six uncultured conjunctival epithelial samples and four out of six primary cultured conjunctival epithelial samples were positive for *TLR9* gene expression. No significant difference in ΔC_t_ was found between cultured and uncultured conjuncitval epithelial cell samples. However, *TLR9* transcripts were not detected in the laser microdissected corneal epithelial cells.

The *TLR4* gene transcript was detected in 5 out of 6 cultured limbal cell samples and 4 out of 6 cultured conjunctival epithelial cell samples. However, it was detected in all uncultured conjunctival epithelial cell samples. The ΔC_t_ for *TLR4* was significantly higher in uncultured conjunctival epithelial cells than the cultured cells. Due to the limited RNA yield from the laser microdissected corneal epithelial cells, the expression of *TLR4* was not analyzed in uncultured corneal epithelial cells.

*TLR7, 8* and *10* gene expression was not detected in any of the limbal or conjunctival epithelial cell samples tested. However, positive *TLR7*, *8*, and *10* transcripts were identified in human spleen cDNA (ΔC_t_ for *TLR7* is 11.5, ΔC_t_ for *TLR8* is 11.5, and ΔC_t_ for *TLR10* is 8.5) [30].

### Western blot analysis of toll-like receptor proteins

**Figure 1:**
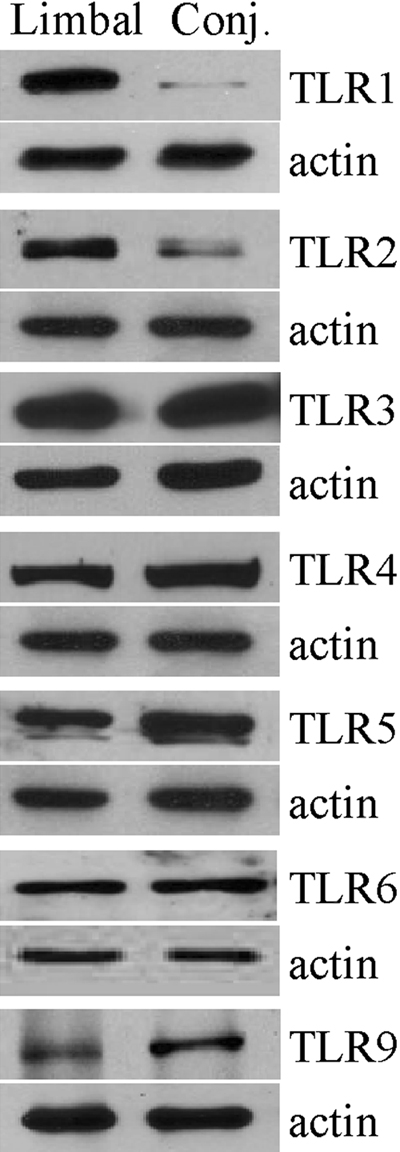
Western blot analysis of toll-like receptor protein expression in cultured human limbal and conjunctival epithelial cells. Three independent samples of primary cultured limbal and conjunctival epithelial cells isolated from different donor tissues with positive identification of each TLR transcript were analyzed (n=3). Cells were lysed in RIPA buffer and 40 μg of total protein was loaded on each lane. The histogram of western blot analysis shows representative results obtained from all samples analyzed. β-Actin was used as the loading control.

In cultured limbal and conjunctival cell samples with positive identification of the respective TLR gene transcripts, specific bands representing the proteins for TLR1 (90 kDa), TLR2 (84 kDa), TLR3 (97 kDa), TLR4 (90 kDa), TLR5 (91 kDa), TLR6 (92 kDa), and TLR9 (116 kDa) were identified in total cell lysates (Figure 1). A significant difference of 16.2 fold in TLR1 and 8.6 fold in TLR2 protein in conjunctival cells was observed when compared to limbal epithelial cells. Other TLR proteins showed no significant differences between the two cell types.

### Immunohistochemistry studies of toll-like receptor distribution

**Figure 2:**
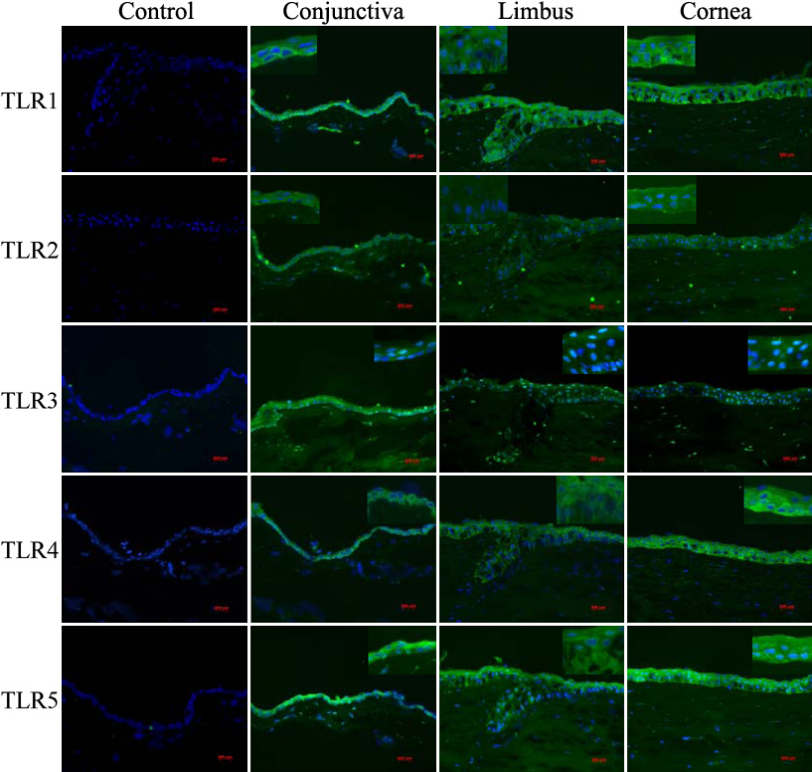
Immunolocalization of toll-like receptors in human corneal, limbal, and conjunctival epithelium. Cryosections of human cornea and conjunctival tissues were incubated with various anti-TLR antibodies and visualized using Alex Fluor 488 conjugated secondary antibodies as described in Methods. Nuclei were stained by DAPI present in the mounting solution. The original pictures were taken at 200X magnification. The insets were taken at 400X magnification.

Immunofluorescence studies of TLR1, 2, 3, 4 and 5 proteins in human corneal, limbal, and conjunctival tissues showed both plasma membrane and cytoplasmic localization of these proteins (Figure 2). The distribution of the above TLR proteins was relatively uniform across the entire corneal epithelium. In limbus, staining of TLR5 was more intense in the upper layers than the basal layer. In conjunctiva, staining for TLR1 and 5 was more intense in the basal layer than in the superficial layers. Some positive results for TLR2, TLR3, and TLR5 were also seen in stromal fibroblasts of the conjunctiva, cornea, and limbus.

Weak fluorescence for TLR9 was also observed in all three cell types (data not shown). However, positive TLR6 binding was not observed in any of the tissue samples tested.

### Ligand-induced NFκB activation in cultured limbal and conjunctival epithelial cells

**Figure 3:**
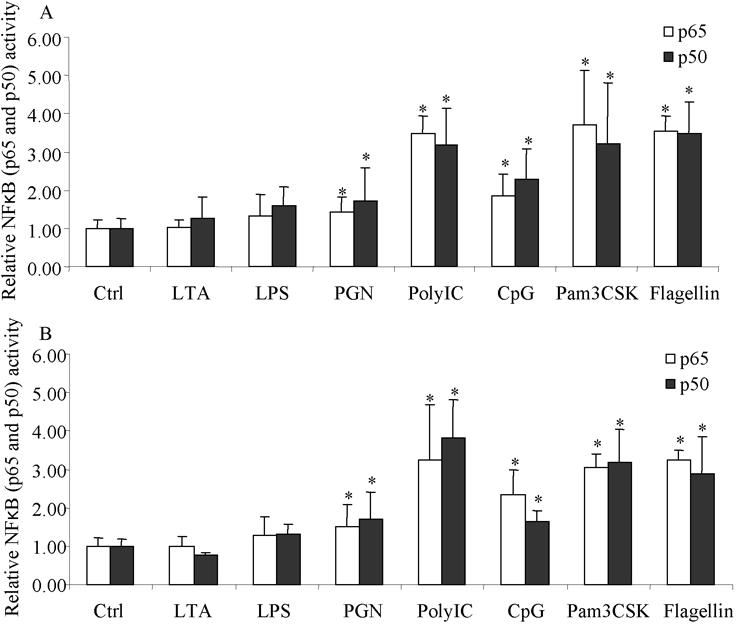
p50 and p65 activities in cultured limbal and conjunctival epithelial cells. Ligand stimulated p50 and p65 activities in cultured limbal (**A**) and conjunctival epithelial cells (**B**) are shown. Eighty percent confluent cells cultured in six well plates (positive for corresponding TLR gene expression) were incubated with different ligands at concentrations indicated in Methods for 8 h before harvesting the cells for the extraction of nuclear proteins. Equal amounts of nuclear proteins were used for the ELISA based analysis of p50 and p65 activities. p50 and p65 activities in control cells (without ligand stimulation) were set as 1 and used to normalize the activities measured in stimulated cells. The open bar represents the p65 subunit and the solid bar represents the p50 subunit. The results show the mean of three independent experiments and the error bar represents the SEM. The asterisk indicates a p<0.05 by ANOVA analysis followed with Fisher LSD test.

To understand if the above identified TLRs were responsive to ligand stimulation, primary cultured limbal and conjunctival epithelial cells were incubated with 1 μg/ml LPS (target TLR4), 1 μg/ml LTA (target TLR2/TLR6 heterodimer); 2 μg/ml flagellin (target TLR5), 10 μg/ml PGN (target TLR2 homodimer), 25 μg/ml poly[I:C] (target TLR3), 250 ng/ml Pam3CSK (target TLR1/2 heterodimer), 1 μM CpG DNA, and CpG control DNA (target TLR9) and the activities of the p60 and p50 subunits of NFκB was measured. Conjunctival and limbal cell samples with positive expression of TLR4 and 9 proteins were used. Increased DNA binding for p50 and p60 was observed at 2 to 5 h after incubation while maximal activities were generally observed 8 h after stimulation for most of the ligands (Figure 3). LTA and LPS, at the concentration range of 10 ng/ml to 10 μg/ml, were not effective in stimulating either p50 or p65 activities in either cell type. Other ligands tested here caused a significant increase in both p50 and p65 activities. The level of response to stimulation was similar for both limbal and conjunctival epithelial cells for a particular ligand. However, the levels varied across the different ligands.

### Ligand-stimulated IL-6 and IL-8 secretion

**Figure 4:**
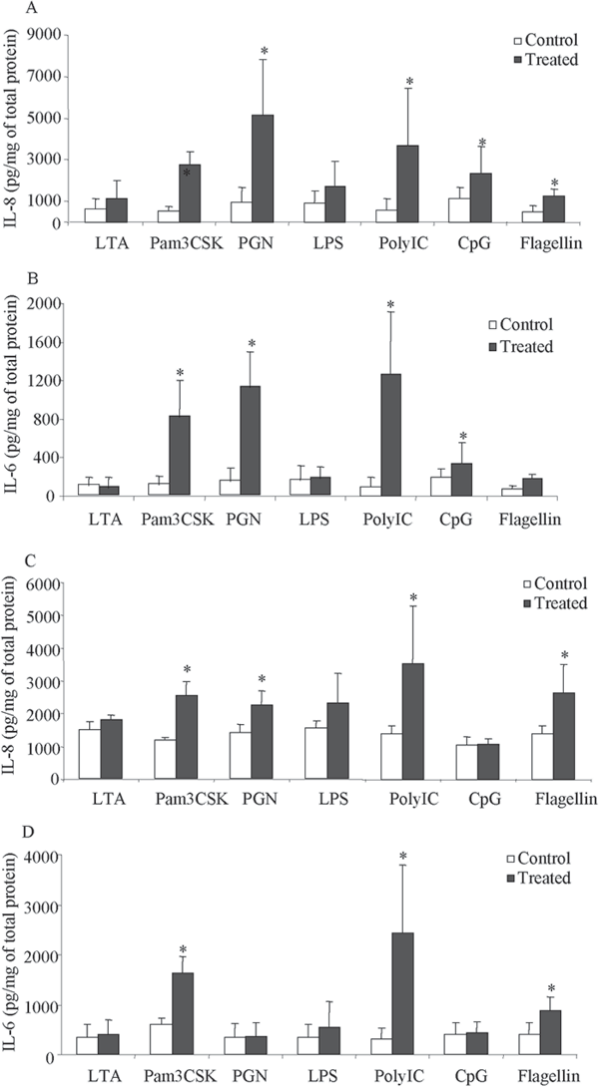
Ligand stimulated IL-6 and IL-8 secretion in cultured limbal and conjunctival epithelial cells. Eighty percent confluent conjunctival (**A** and **B**) and limbal (**C** and **D**) cells positive of corresponding TLR gene expression were incubated with different ligands at the concentrations indicated in Methods. Culture medium was collected 24 h later. IL-8 (**A** and **C**) and IL-6 (**B** and **D**) protein levels were measured by ELISA. Data represent the mean of four to five independent experiments and the error bar represents the SEM. The concentration of IL-6 and IL-8 was corrected by total protein amount in each well at the time of harvesting the supernatant. Significance compared to the controls by ANOVA and Fisher LSD analysis at a level of p<0.05 is shown by the asterisk. For CpG stimulated cells, the control refers to the CpG control DNA stimulated cells.

The levels of IL-6 and IL-8 in cell culture medium 24 h after ligand stimulation are shown in Figure 4. Pam3CSK, poly(I:C), and flagellin caused a significant increase in IL-6 and IL-8 proteins in both conjunctival and limbal epithelial cells. PGN and CpG DNA induced a significant increase of both IL-6 and IL-8 in conjunctival epithelial cells. PGN-stimulated increase of IL-6 and IL-8 was observed in three out of four different primary cultured limbal epithelial cell samples. Marginal CpG-stimulated increases of IL-6 and IL-8 were observed in two out of four different primary cultured limbal epithelial cell samples.

Similar to the lack of activation of NFκB, neither LTA nor LPS elicited a significant increase in IL-6 or IL-8 production in either limbal or conjunctival cells. The lack of LPS-induced IL-6 and IL-8 production was verified using LPS obtained from two different bacteria strains (*Pseudomonas aeruginosa* and *E. Coli.*), at concentrations up to 10 μg/ml.

### Expression and contribution of MD2, CD14, and LBP in limbal and conjunctival epithelial cells

Controversial results were reported on the existence of LPS-stimulated inflammatory responses in cultured human corneal epithelial cells [16,17,22]. In addition to TLR4, MD2, CD14, and LBP are part of the LPS recognition complex. It was reported that tear LBP and CD14 proteins were needed for the positive LPS-induced cytokine secretion in corneal epithelial cells [22]. Expression of MD2 (ΔC_t_ for conjunctival: 13.69±2.80, ΔC_t_ for limbal: 9.23±1.40, n=6) of and CD14 (ΔC_t_ for conjunctival: 9.22±1.25, ΔC_t_ for conjunctival: 0.28±0.44, n=6) was confirmed in all cultured cells. However, the LBP gene transcript was not detected in any of the cultured conjunctival cell samples while it was detected in two out of six samples of cultured limbal epithelial cells.

To make sure that the lack of a LPS-induced response was not due to an insufficient amount of CD14 or LBP proteins in the cell culture media, we added carrier-free recombinant human CD14 (500 ng/ml) and LBP (150 ng/ml) proteins to the culture medium separately and in combination. Furthermore, we used sensitive real-time PCR analysis to monitor the changes of IL-6 and IL-8 gene expression in these cells at 2, 4, 8, and 16 h after the combined stimulation (control; 1 μg/ml LPS; 1 μg/ml LPS with 500 ng/ml CD14; 1 μg/ml LPS with 150 ng/ml of LBP; 1 μg/ml LPS with 500 ng/ml CD14, and 150 ng/ml LBP). However, we were not able to detect any change in IL-6 or IL-8 gene expression when compared to controls (data not shown).

## Discussion

The ocular surface is covered with epithelial cells with three specific phenotypes: corneal, conjunctival, and limbal. It is anticipated that the differences in the cell biology and in the responses to inflammation in these cells would be reflected in their expression of TLRs and responses to pathogen pattern molecule stimulation. Although TLR2, 3, 4, and 5 were previously found in corneal epithelial cells [14,16-18], only one study reported the expression of TLR2, 4, and 9 in human conjunctival epithelial cells and the functional analysis of these TLRs in conjunctival epithelial cells was lacking [13,31]. The distribution and function of TLRs in limbal epithelial cells were unknown. In the present experiments, the expression and function of TLR1-10 in both primary cultured and uncultured conjunctival epithelial cells as well as primary cultured limbal epithelial cells were studied. We consistently found the expression of *TLR1, 2, 3*, and *5* genes and the protein in cultured limbal and conjunctival cell sample. Stimulation with specific ligands targeted to the TLR1/2 heterodimer, TLR3, and TLR5 for the cultured limbal and conjunctival epithelial cells showed an increase in NFκB activity as well as increased IL-6 and IL-8 secretion in the culture medium. The results strongly suggest that the expressed receptors, TLR1, 2, 3, and 5, are functional in these cells.

Previously, TLR5- and TLR3-mediated pro-inflammatory responses were reported by other groups in human corneal epithelial cells [14,18]. A recent study from Cook et al. [31] reported that human conjunctival epithelial cells responded to cell wall extract of *S. aureus* with increased TNFα and IL-8 secretion. Although no distinction was made among the TLR dimers in the report, TLR1/TLR2 heterodimer was most likely responsible for the recognition of the cell wall extract of *S. aureus*. Similarly, using Pam3Cys, Johnson et al reported positive cytokine responses in mouse corneal epithelial cells [20]. Taken together, these studies suggest that TLR1/TLR2, TLR3, and TLR5 activate intracellular programs with downstream effector complexes in ocular surface epithelial cells.

The expression and function of TLR6 and 9 in conjunctival and limbal epithelial cells are more complicated. While all cell samples were positive for *TLR6* gene expression, four out of 11 conjunctival epithelial cell samples tested (cultured and uncultured combined) were negative for *TLR9* gene transcripts. Although all cultured limbal epithelial cell samples were positive for *TLR9* expression, none of the corneal epithelial cell samples showed positive TLR9 expression. However, this could be due to the limited amount of cDNA available from laser microdissected corneal epithelial cells. High ΔC_t_ was observed for both gene transcripts in cultured and uncultured cell samples, which indicated a low copy number of the transcripts. Furthermore, LTA targeting of the TLR2/6 heterodimer did not cause changes of NFκB activity or IL-6/IL-8 secretion in limbal or conjunctival epithelial cells. Similar unresponsiveness to LTA was also observed in cultured normal intestinal epithelial cells [32]. Additionally, CpG DNA-stimulated responses in limbal and conjunctival epithelial cells were not uniform among different samples. Since different TLR proteins share the same intracellular signaling network, we believe that the low abundance of the TLR6 and 9 proteins are a likely reason for the lack of ligand-induced cellular responses in these samples.

The expression of TLR4 has been reported in corneal and conjunctival epithelial cells [13,16,17]. While LPS-stimulated responses were not studied in conjunctival epithelial cells, controversial results were reported on LPS-induced inflammatory responses in human corneal epithelial cells [16,17]. An early study from Song et al. [16] showed a clear LPS-stimulated, CD14 antibody-inhibited IL-6 and IL-8 secretion in a human corneal epithelial cell line. However, a later report from Ueta et al. [17] showed that LPS incubation had no effect on TLR4 surface distribution or IL-6 and IL-8 secretion in either primary cultured or immortalized human corneal epithelial cells. No changes in cytokine production or NFκB activity were observed even when chromogen-conjugated LPS molecules were injected into these cells. A more recent report showed that additional LBP and CD14 were required for LPS stimulated IL-6 and IL-8 secretion in corneal epithelial cells [22]. The lack of LPS-induced inflammatory responses was also observed in epithelial cells originating from other mucosal surfaces such as human intestinal and oral mucosa under normal culture conditions [33-35]. We found *TLR4* transcripts in all the uncultured conjunctival epithelial cell samples. The expression of *TLR4* was significantly reduced in cultured conjunctival and limbal epithelial cells and was even undetectable in some of the cultured cell samples. However, even in cells with positive TLR4 gene and protein expression, we were not able to detect changes in NFκB activity or IL-6, IL-8 secretion upon LPS stimulation at various concentrations. We further demonstrated that the lack of a response was not due to the absence of CD14 or LBP proteins. Our results suggest that a TLR4-mediated LPS-induced proinflammatory response does not exist in primary cultured human limbal or conjunctival epithelial cells. This corroborates the conclusions drawn by Ueta et al. [17] from their studies on corneal epithelial cells as well as the results from other groups working on intestinal and oral mucosal epithelial cells [32,33,35]. It was reported that priming with cytokines such as IFNγ and TNFα renders mucosal and intestinal epithelial cells responsive to LPS stimulation [35,36]. Whether ocular surface epithelial cells need similar priming in order to respond to LPS is yet to be determined.

The present study showed that human conjunctival and limbal epithelial cells are protected by TLRs, which recognize pattern molecules from a broad spectrum of pathogens. However, differences in the abundance of individual TLR transcripts and the responses to ligand stimulation exist in both limbal and conjunctival epithelial cells. Such differences may reflect a selective requirement of different TLRs in order to maintain a delicate balance between immune tolerance for ocular surface commensal bacteria and the protection against microorganism invasion. However, one has to be careful when applying the results obtained from cultured cells to in vivo conditions. This is evidenced by the significantly higher abundance of *TLR1, 2, 3, 4, 5*, and *6* gene transcripts in uncultured conjunctival epithelial cells than the cultured counterpart. Therefore, it is possible the TLR-mediated inflammatory responses through ocular surface epithelial cells are more robust than what we observed in cell culture simply due to the higher levels of the individual TLR gene expression. Furthermore, the lack of LPS- and LTA-induced inflammatory responses may not hold true in vivo. Rapid and robust LPS-induced inflammatory responses were observed in mouse cornea when the integrity of corneal epithelium were surgically breached and the epithelial cell TLR4-mediated inflammatory response was concluded [20]. Furthermore, the differences in the transcripts levels clearly indicate that the expression of TLR genes are susceptible to changes of the extracellular environment. This implies that the contribution of the epithelial cell TLRs may further vary under different physiological and pathological conditions of the ocular surface. Regulation of TLR4 expression by IFNγ and TNFα was reported in human intestinal epithelial cells and oral mucosal epithelial cells [33,35]. Although the expression of TLR7, 8 and 10 was not detected in the current study, it is still possible that the expression of these genes and proteins can be induced in vivo. For example, it was recently found that herpes simplex virus induced TLR7 expression in human corneal cells [19]. Studies on the regulation of TLR gene expression and TLR-mediated inflammatory responses are needed to better understand the role of epithelial cell-borne TLR in the protection of ocular surface against pathogen invasion.

In summary, the present study demonstrated the expression of multiple TLRs in human conjunctival and limbal epithelial cells. However, the abundance and the corresponsive ligand-induced inflammatory responses are different among these TLRs in both limbal and conjunctival epithelial cells. Our study also suggested that the expression of TLRs is susceptible to the changes of the extracellular environment. While the results clearly showed the active role of human ocular surface epithelial cells in TLR-mediated innate immune responses against microorganism invasion, it also implied that a delicate balance exists between the desired immune tolerance and the dynamic regulation of TLR expression and function in conjunctival and limbal epithelial cells.

## References

[1] Lemaitre B Nicolas E Michaut L Reichhart JM Hoffmann JA The dorsoventral regulatory gene cassette spatzle/Toll/cactus controls the potent antifungal response in Drosophila adults. Cell 1996 86 973 83 8808632 10.1016/s0092-8674(00)80172-5

[2] Aderem A Ulevitch RJ Toll-like receptors in the induction of the innate immune response. Nature 2000 406 782 7 10963608 10.1038/35021228

[3] Hayashi F Smith KD Ozinsky A Hawn TR Yi EC Goodlett DR Eng JK Akira S Underhill DM Aderem A The innate immune response to bacterial flagellin is mediated by Toll-like receptor 5. Nature 2001 410 1099 103 11323673 10.1038/35074106

[4] Ozinsky A Underhill DM Fontenot JD Hajjar AM Smith KD Wilson CB Schroeder L Aderem A The repertoire for pattern recognition of pathogens by the innate immune system is defined by cooperation between toll-like receptors. Proc Natl Acad Sci USA 2000 97 13766 71 11095740 10.1073/pnas.250476497 PMC17650

[5] Miyake K Endotoxin recognition molecules, Toll-like receptor 4-MD-2. Semin Immunol 2004 16 11 6 14751758 10.1016/j.smim.2003.10.007

[6] Pugin J Schurer-Maly CC Leturcq D Moriarty A Ulevitch RJ Tobias PS Lipopolysaccharide activation of human endothelial and epithelial cells is mediated by lipopolysaccharide-binding protein and soluble CD14. Proc Natl Acad Sci USA 1993 90 2744 8 7681988 10.1073/pnas.90.7.2744 PMC46172

[7] Tobias PS Soldau K Gegner JA Mintz D Ulevitch RJ Lipopolysaccharide binding protein-mediated complexation of lipopolysaccharide with soluble CD14. J Biol Chem 1995 270 10482 8 7537731 10.1074/jbc.270.18.10482

[8] Morr M Takeuchi O Akira S Simon MM Muhlradt PF Differential recognition of structural details of bacterial lipopeptides by toll-like receptors. Eur J Immunol 2002 32 3337 47 12432564 10.1002/1521-4141(200212)32:12<3337::AID-IMMU3337>3.0.CO;2-#

[9] Takeuchi O Kawai T Muhlradt PF Morr M Radolf JD Zychlinsky A Takeda K Akira S Discrimination of bacterial lipoproteins by Toll-like receptor 6. Int Immunol 2001 13 933 40 11431423 10.1093/intimm/13.7.933

[10] Hajjar AM O'Mahony DS Ozinsky A Underhill DM Aderem A Klebanoff SJ Wilson CB Cutting edge: functional interactions between toll-like receptor (TLR) 2 and TLR1 or TLR6 in response to phenol-soluble modulin. J Immunol 2001 166 15 9 11123271 10.4049/jimmunol.166.1.15

[11] Takeda K Akira S TLR signaling pathways. Semin Immunol 2004 16 3 9 14751757 10.1016/j.smim.2003.10.003

[12] Zhang G Ghosh S Toll-like receptor-mediated NF-kappaB activation: a phylogenetically conserved paradigm in innate immunity. J Clin Invest 2001 107 13 9 11134172 10.1172/JCI11837 PMC198554

[13] Bonini S Micera A Iovieno A Lambiase A Bonini S Expression of Toll-like receptors in healthy and allergic conjunctiva. Ophthalmology 2005 112 1528discussion1548-9 16023216 10.1016/j.ophtha.2005.04.009

[14] Ueta M Hamuro J Kiyono H Kinoshita S Triggering of TLR3 by polyI:C in human corneal epithelial cells to induce inflammatory cytokines. Biochem Biophys Res Commun 2005 331 285 94 15845391 10.1016/j.bbrc.2005.02.196

[15] Kumar A Zhang J Yu FS Toll-like receptor 3 agonist poly(I:C)-induced antiviral response in human corneal epithelial cells. Immunology 2006 117 11 21 16423036 10.1111/j.1365-2567.2005.02258.x PMC1782193

[16] Song PI Abraham TA Park Y Zivony AS Harten B Edelhauser HF Ward SL Armstrong CA Ansel JC The expression of functional LPS receptor proteins CD14 and toll-like receptor 4 in human corneal cells. Invest Ophthalmol Vis Sci 2001 42 2867 77 11687531

[17] Ueta M Nochi T Jang MH Park EJ Igarashi O Hino A Kawasaki S Shikina T Hiroi T Kinoshita S Kiyono H Intracellularly expressed TLR2s and TLR4s contribution to an immunosilent environment at the ocular mucosal epithelium. J Immunol 2004 173 3337 47 15322197 10.4049/jimmunol.173.5.3337

[18] Zhang J Xu K Ambati B Yu FS Toll-like receptor 5-mediated corneal epithelial inflammatory responses to Pseudomonas aeruginosa flagellin. Invest Ophthalmol Vis Sci 2003 44 4247 54 14507868 10.1167/iovs.03-0219

[19] Li H Zhang J Kumar A Zheng M Atherton SS Yu FS Herpes simplex virus 1 infection induces the expression of proinflammatory cytokines, interferons and TLR7 in human corneal epithelial cells. Immunology 2006 117 167 76 16423052 10.1111/j.1365-2567.2005.02275.x PMC1782219

[20] Johnson AC Heinzel FP Diaconu E Sun Y Hise AG Golenbock D Lass JH Pearlman E Activation of toll-like receptor (TLR)2, TLR4, and TLR9 in the mammalian cornea induces MyD88-dependent corneal inflammation. Invest Ophthalmol Vis Sci 2005 46 589 95 15671286 10.1167/iovs.04-1077

[21] Huang X Barrett RP McClellan SA Hazlett LD Silencing Toll-like receptor-9 in Pseudomonas aeruginosa keratitis. Invest Ophthalmol Vis Sci 2005 46 4209 16 16249500 10.1167/iovs.05-0185

[22] Blais DR Vascotto SG Griffith M Altosaar I LBP and CD14 secreted in tears by the lacrimal glands modulate the LPS response of corneal epithelial cells. Invest Ophthalmol Vis Sci 2005 46 4235 44 16249503 10.1167/iovs.05-0543

[23] Kumar A Zhang J Yu FS Innate immune response of corneal epithelial cells to Staphylococcus aureus infection: role of peptidoglycan in stimulating proinflammatory cytokine secretion. Invest Ophthalmol Vis Sci 2004 45 3513 22 15452057 10.1167/iovs.04-0467 PMC2666393

[24] Kumar MV Nagineni CN Chin MS Hooks JJ Detrick B Innate immunity in the retina: Toll-like receptor (TLR) signaling in human retinal pigment epithelial cells. J Neuroimmunol 2004 153 7 15 15265658 10.1016/j.jneuroim.2004.04.018 PMC7119465

[25] Aliprantis AO Yang RB Mark MR Suggett S Devaux B Radolf JD Klimpel GR Godowski P Zychlinsky A Cell activation and apoptosis by bacterial lipoproteins through toll-like receptor-2. Science 1999 285 736 9 10426996 10.1126/science.285.5428.736

[26] Hemmi H Kaisho T Takeuchi O Sato S Sanjo H Hoshino K Horiuchi T Tomizawa H Takeda K Akira S Small anti-viral compounds activate immune cells via the TLR7 MyD88-dependent signaling pathway. Nat Immunol 2002 3 196 200 11812998 10.1038/ni758

[27] Chan CM Liu YP Tan DT Ocular surface changes in pterygium. Cornea 2002 21 38 42 11805505 10.1097/00003226-200201000-00009

[28] Li J Raghunath M Tan D Lareu RR Chen Z Beuerman RW Defensins HNP1 and HBD2 stimulation of wound-associated responses in human conjunctival fibroblasts. Invest Ophthalmol Vis Sci 2006 47 3811 9 16936092 10.1167/iovs.05-1360

[29] Ti SE Anderson D Touhami A Kim C Tseng SC Factors affecting outcome following transplantation of ex vivo expanded limbal epithelium on amniotic membrane for total limbal deficiency in rabbits. Invest Ophthalmol Vis Sci 2002 43 2584 92 12147589

[30] Nishimura M Naito S Tissue-specific mRNA expression profiles of human toll-like receptors and related genes. Biol Pharm Bull 2005 28 886 92 15863899 10.1248/bpb.28.886

[31] Cook EB Stahl JL Esnault S Barney NP Graziano FM Toll-like receptor 2 expression on human conjunctival epithelial cells: a pathway for Staphylococcus aureus involvement in chronic ocular proinflammatory responses. Ann Allergy Asthma Immunol 2005 94 486 97 15875531 10.1016/S1081-1206(10)61120-9

[32] Melmed G Thomas LS Lee N Tesfay SY Lukasek K Michelsen KS Zhou Y Hu B Arditi M Abreu MT Human intestinal epithelial cells are broadly unresponsive to Toll-like receptor 2-dependent bacterial ligands: implications for host-microbial interactions in the gut. J Immunol 2003 170 1406 15 12538701 10.4049/jimmunol.170.3.1406

[33] Abreu MT Vora P Faure E Thomas LS Arnold ET Arditi M Decreased expression of Toll-like receptor-4 and MD-2 correlates with intestinal epithelial cell protection against dysregulated proinflammatory gene expression in response to bacterial lipopolysaccharide. J Immunol 2001 167 1609 16 11466383 10.4049/jimmunol.167.3.1609

[34] Rakoff-Nahoum S Paglino J Eslami-Varzaneh F Edberg S Medzhitov R Recognition of commensal microflora by toll-like receptors is required for intestinal homeostasis. Cell 2004 118 229 41 15260992 10.1016/j.cell.2004.07.002

[35] Uehara A Sugawara S Takada H Priming of human oral epithelial cells by interferon-gamma to secrete cytokines in response to lipopolysaccharides, lipoteichoic acids and peptidoglycans. J Med Microbiol 2002 51 626 34 12171292 10.1099/0022-1317-51-8-626

[36] Abreu MT Arnold ET Thomas LS Gonsky R Zhou Y Hu B Arditi M TLR4 and MD-2 expression is regulated by immune-mediated signals in human intestinal epithelial cells. J Biol Chem 2002 277 20431 7 11923281 10.1074/jbc.M110333200

